# Exploring the limitations of mitochondrial dye as a genuine horizontal mitochondrial transfer surrogate

**DOI:** 10.1038/s42003-024-05964-6

**Published:** 2024-03-07

**Authors:** Chuanfang Chen, Haige Li, Jia Zhang, Shih-Chin Cheng

**Affiliations:** 1 State Key Laboratory of Cellular Stress Biology, School of Life Sciences, Faculty of Medicine and Life Sciences, Xiamen University; Xiamen, Fujian, 361102 China; 2 Department of Gastroenterology, The National Key Clinical Specialty, Zhongshan Hospital of Xiamen University, School of Medicine, Xiamen University, Xiamen, Fujian, 361004 China; 3 Department of Digestive Disease, School of Medicine, Xiamen University, Xiamen, Fujian, 361004 China

**Keywords:** Mitochondria, Cell biology

## Abstract

Rosamine-based mitochondrial dyes, such as Mitotracker Red, have commonly been employed to visualize mitochondrial localization within cells due to their preferential accumulation in organelles with membrane potential. Consequently, Mitotracker Red has often served as a surrogate indicator for tracking mitochondrial movement between neighboring cells. However, it is important to note that the presence of membrane potential in the cell membrane and other organelles may lead to the non-specific partial enrichment of Mitotracker Red in locations other than mitochondria. This study comprehensively investigates the reliability of mitochondrial dye as a marker for studying horizontal mitochondrial transfer (HMT). By meticulous replicating of previous experiments and comparing the efficiency of mitochondrial dye transfer with that of mito-targeted GFP, our findings confirm that HMT occurs at significantly lower efficiency than previously indicated by Mitotracker dye. Subsequent experiments involving mitochondria-deficient cells robustly demonstrates the non-specificity of mitochondrial dye as indicator for mitochondria. We advocate for a thorough reevaluation of existing literature in this field and propose exploration of alternative techniques to enhance the investigation of HMT. By addressing these pivotal aspects, we can advance our understanding of cellular dynamics and pave the way for future explorations in this captivating field.

## Abstract (web summary)

Evidence underscores the non-specificity of mitochondrial dye in assessing horizontal mitochondrial transfer, suggesting that alternative approaches should be considered for gaining nuanced insights into intercellular mitochondrial dynamics.

## Introduction

Mitochondria play a vital role in diverse cellular activities due to their involvement in energy metabolism and signaling pathways. Over the past two decades, numerous studies have unveiled an intriguing phenomenon called horizontal mitochondria transfer (HMT), wherein mitochondria exhibit the ability to migrate between cells^1^. HMT has been increasingly reported in various cell types, with particular focus on mesenchymal stem cells (MSCs)^2–5^. For example, MSCs have been found to donate mitochondria to alveolar adenocarcinoma cells^2^, cardiomyoblasts^6^, kidney renal tubular cells^7^, osteosarcoma cells^8^, vascular smooth muscle cells^9^, epithelial cells^3,5^, neuronal cells^4^, macrophages^10^, astrocytes^4^, and T cells^11^. Other cell types have also been reported as mitochondria donors, including neuronal cells^12^, kidney renal tubular cells^7^, cardiomyoblasts^13^, macrophages^14^, fibroblast^15^, astrocytes^16^, platelets^17^, and T cells^18^. Multiple mechanisms have been described for HMT, such as tunneling nanotubes (TNT)^7^, gap junction^19^, cell fusion^20^, endocytosis^21^, cell–cell contact^22^, and secreted vesicles^23^. HMT has been widely identified both in vitro^24,25^ and in vivo^26–28^.

One noteworthy observation in the relevant literature is the prevalent use of mitochondria labeling dye to indicate HMT between cells (Supplementary Data 1). Mitochondria transfer positively charged protons across the inner membrane resulting in a net internal negative charge, known as the mitochondrial membrane potential (MMP), typically maintained around −180 mV in healthy mitochondria^29^. Dyes commonly used for mitochondria staining in HMT studies, including rosamine-based, carbocyanine-based, and rhodamine-based probes, possess a positive charge^30^. For instance, rosamine-based probes encompass a series of Mitotracker CMTMRos/CM-H2TMRos/CMXRos/CM-H2XRos, while carbocyanine-based probes include Mitotracker FM. Rosamine-based mitotracker dyes contain a mild thiol-reactive chloromethyl moiety and accumulate in mitochondria depending on MMP^31–34^, enabling dyes to react with thiols on proteins and peptides on mitochondria, thereby emitting fluorescence indicative of mitochondrial morphology^30^. However, it is important to consider that thiol-containing peptides are present in numerous proteins, including those located in mitochondria^35^, endoplasmic reticulum^36^, Golgi apparatus^37^, and cell membrane^38^. The existence of membrane potential relies on the ion concentration gradient on both sides of the membrane, which can be found in membrane-containing organelles such as the mitochondria^29^, endoplasmic reticulum^39^, Golgi apparatus^40^, lysosome^41^, and even the cell membrane^42^. While healthy mitochondria exhibit the highest membrane potential, cells labeled with Mitotracker dyes predominantly display clear mitochondrial morphology. However, it cannot be ruled out that mitotracker might also be attracted and accumulated by other membrane structures, which often results in a weak signal that has often been disregarded as background noise. In early literature utilizing mitotracker as a mitochondria indicator, images depicted not only a strong signal in mitochondria but also a weak signal around the cell, resembling a cell membrane^30^. The weak signal in the cell membrane was disregarded as background noise. Subsequent publications predominantly employed mitotracker staining for functional experiments both in vitro and in vivo (Supplementary Data 1), overlooking the potential non-specificity of mitotracker. These concerns raise doubts regarding the authenticity and reliability of studies investigating HMT solely based on mitotracker labeling, necessitating further scrutiny to avoid misleading future research endeavors.

In the present study, we aim to provide a comprehensive summary and discussion of the non-specificity of mitochondria dyes in the HMT study. Specifically, we compare the transfer rate of mitochondria dyes with that of proteins specifically localized to mitochondria, highlighting the significantly lower efficiency of protein-based markers compared to dyes. Furthermore, our results demonstrate the transfer of dyes between mitochondria-depleted cells and wild-type cells, suggesting the potential non-specific nature of mitochondrial dye transfer. Finally, we observe the transfer of various mitochondrial dyes from red blood cells (RBCs), which do not contain mitochondria, to recipient 293T cells, robustly establishing that mitochondrial dye transfer does not equal to actual HMT.

## Results

### Mitotracker Red can transfer between various type of cells

**Fig. 1:**
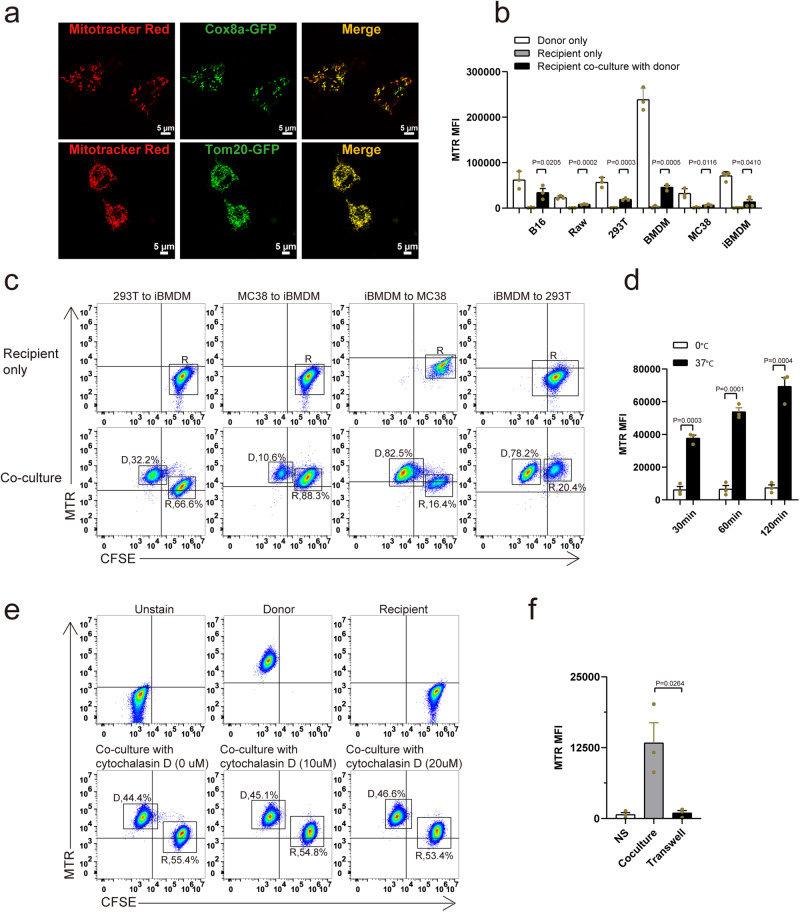
Mitochondrial dye can transfer between cells. **a** Confocal image showed co-localization of overexpressed mito-target GFP and Mitotracker Red dye in iBMDM cells. **b**, **c** Cytometry analysis of recipient cells received MTR signal between same kind (**b**) or different kind (**c**) of cells (D, donor; R, recipient; *n* = 3 biologically independent experiments). **d**–**f** Cytometry analysis of iBMDM recipient cells received MTR signal at different temperatures at different times (**d**), cytochalasin D pretreated (**e**), and transwell condition (**f**, *n* = 3 biologically independent experiments).

To validate previous findings on HMT, we successfully replicated experiments using Mitotracker Red (MTR) to label mitochondria. Immortalized bone marrow-derived cell (iBMDM) cells overexpressing mitochondrial-targeted GFP (COX8a signal peptide or whole TOM20 CDS region fused with GFP, referred to as mito-targeted GFP) were labeled with MTR, and confocal images demonstrated co-localization of mito-targeted GFP and MTR signals within mitochondria (Fig. 1a). Next, we extended HMT investigations to various cell types, including both published (macrophage, B16 cells, 293T cells)^10,26,43^ and unpublished (MC38 cells) cells. MTR-labeled donor cells were co-cultured with recipient cells, co-cultured recipients exhibited higher MTR signal compared to non-co-cultured recipients, confirming the transfer of MTR signal from donor cells to recipients (Fig. 1b). In line with previous literature^7,44,45^, MTR signal was also observed to transfer between different cell types (Fig. 1c).

To address concerns about potential dye leakage, we meticulously washed the MTR-labeled donor cells with fresh medium five times and collected washed buffer. We co-cultured recipient cells with washed buffer, and results showed that residual dye was gradually reduced with the increase in washing times, with almost no dye remaining after the fourth wash, which ruled out dye leakage as the cause of MTR signal transfer (Supplementary Fig. 1A).

Since the formation of tunneling nanotubes (TNT) has been identified as one of the primary routes for HMT, we explored whether MTR signal transfer might be influenced by temperature. TNT formation is known to depend on actin polymerization and ATP hydrolysis, and its efficiency is affected by temperature. To investigate this, we co-cultured iBMDM cells at two distinct temperatures: 37 and 0 °C, for varying time intervals. Our data revealed that MTR signal transfer occurred rapidly at 37 °C but was significantly inhibited when cells were co-cultured at 0 °C (Fig. 1d and Supplementary Fig. 1B). Since numerous cellular processes are arrested at 0 °C, we proceeded to inhibit TNT formation using an actin polymerization inhibitor. Notably, contrary to what has been reported in previous studies^3,7,9^, we consistently observed the detection of the MTR transfer signal in recipient cells, regardless of the concentration of cytochalasin D used (Fig. 1e). Moreover, we further examined whether direct cell–cell contact was indeed necessary for the transfer of MTR signals. To test this hypothesis, we implemented a transwell system, physically separating the donor cells from the recipient cells. Our results provide clear evidence that cell–cell contact was necessary for MTR signal transfer in our system in vitro (Fig. 1f, Supplementary Fig. 1C, and Supplementary Video).

### Mitochondria dye transfer was not equal to mitochondria transfer

**Fig. 2:**
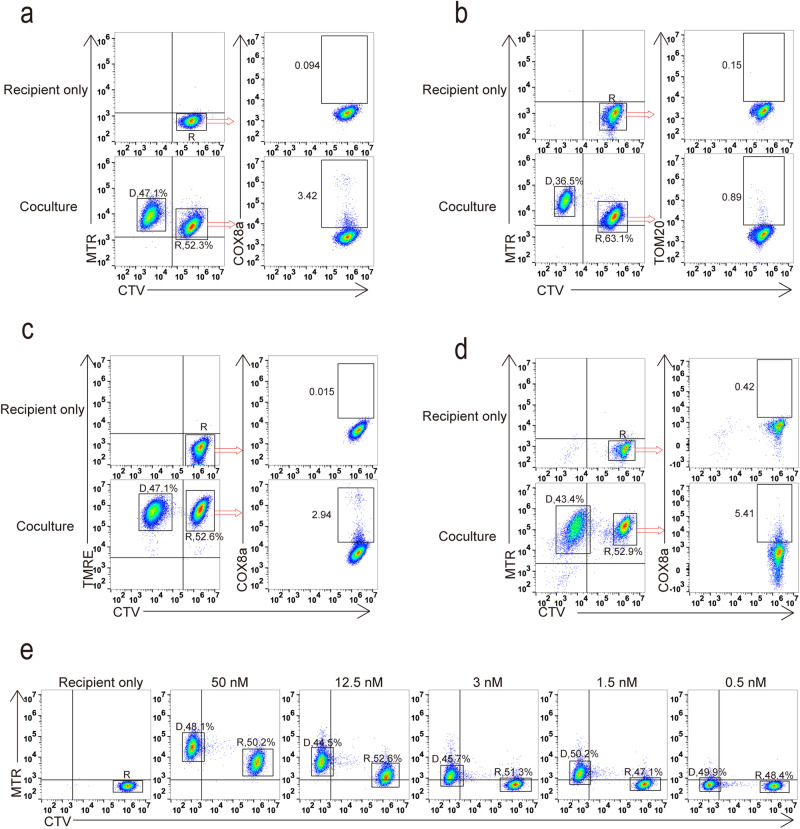
Mitochondria dye transfer was not equal to mitochondria transfer. **a**, **b** Cytometry analysis of iBMDM recipient cells received MTR signal compared to overexpressed COX8a tagged GFP (a) or TOM20-GFP fusion protein signal (**b**). **c** Cytometry analysis of iBMDM recipient cells received TMRE signal compared to COX8a tagged GFP signal. **d** Cytometry analysis of 293 T recipient cells received MTR signal compared to COX8a tagged GFP signal from MSCs. **e** Cytometry analysis of iBMDM recipient cells received MTR signal at different concentrations. (D, donor; R, recipient).

As we previously suspected, MTR could be accumulated not only in mitochondria, but also in other membranous organelles possibly. To investigate the disparity between MTR signal delivery and HMT, we co-cultured MTR-labeled iBMDM donor cells overexpressing COX8a-GFP with CTV-labeled recipient cells. Flow cytometry analysis demonstrated that most recipient cells received the MTR signal rather than the COX8a-GFP signal from donor cells (Fig. 2a and Supplementary Fig. 2A). Similar results were obtained when donor cells overexpressing TOM20-GFP fusion protein as a marker for mitochondria. The efficiency of TOM20-GFP transfer was significantly lower than that of the MTR signal (Fig. 2b and Supplementary Fig. 2B). Additionally, we also compared the transfer efficiency of another mitochondria dye, TMRE, with the mito-targeted GFP signal. We employed TMRE to label donor cells overexpressing COX8a-GFP and then co-cultured with CTV-labeled recipients. The results indicated that only a small proportion of recipient cells received COX8a-GFP signal, while nearly all recipient cells received TMRE signal (Fig. 2c and Supplementary Fig. 2C). To further validate these observations, we isolated mesenchymal stem cells (MSCs), the most well-characterized mitochondria donor cells, from mouse epididymal adipose tissue. We labeled MSCs overexpressing COX8a-GFP with MTR, and co-cultured it with CTV-labeled 293T recipient cells. Flow cytometry analysis confirmed that the MTR signal could be transferred from MSCs to recipient cells, while the COX8a-GFP signal did not exhibit comparable transfer efficiency (Fig. 2d and Supplementary Fig. 2D). Intriguingly, we noticed an inconsistent result wherein the efficiency of dye transfer decreased as the MTR concentration in the donor staining decreased. When donor cells were labeled with MTR at 50 nM concentration, both the donor and recipient cells displayed obvious mitochondrial staining signal. However, this consistency was disrupted when the concentration dropped to 1.5 nM. At 1.5 nM, the donor cells completely separated from the unstained population, while the recipient cells exhibited minimal mitochondrial signal (Fig. 2e).

While these findings raise important questions, it is essential to acknowledge that further scrutiny is warranted to fully comprehend the intricacies of this phenomenon. The possibility that HMT, as indicated by mitochondrial dyes, may not exclusively represent genuine HMT but could instead be attributed to the transfer of the dye itself calls for cautious consideration. Consequently, a critical reevaluation of numerous studies employing mitochondrial dyes to indicate HMT between cells and subsequent functional verifications is imperative to avoid drawing potentially misleading conclusions in future research endeavors.

### Mitotracker Red can transfer between mito-depleted and WT cells

**Fig. 3:**
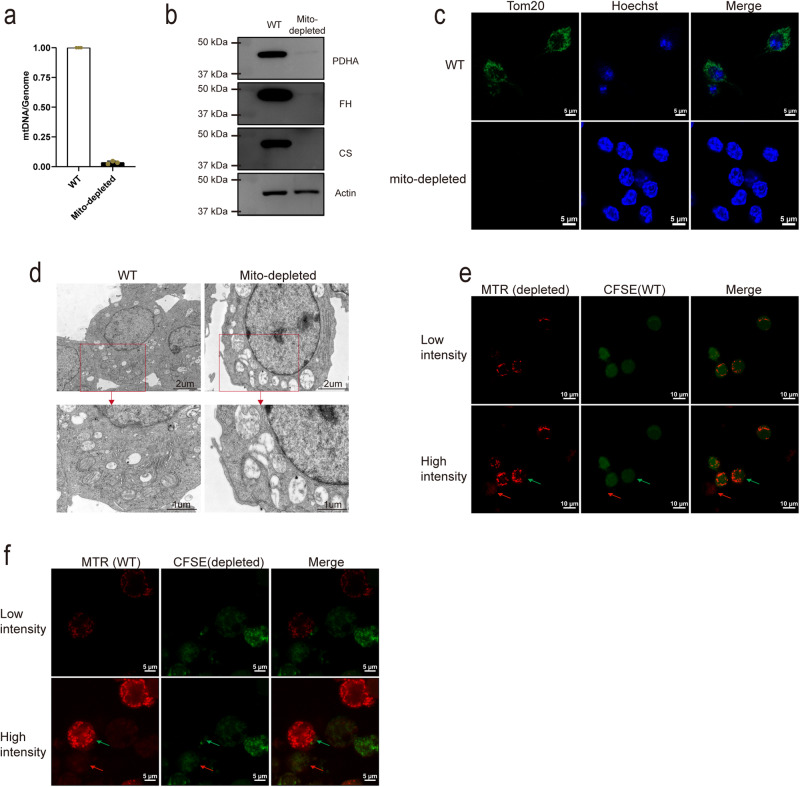
Mitotracker dye can transfer between mito-depleted and WT cells. **a**, **b** Quantifications of mtDNA content (a, *n* = 3 biologically independent experiments) and mitochondrial located proteins (**b**, blots were tested from separated gels loading the same volume of sample) in WT and CCCP-treated mito-depleted Raw 264.7 cells. **c**, **d** Immunofluorescence staining of antibody against TOM20 (**c**) and TEM analysis of mitochondrial number and morphology (**d**) in WT and mito-depleted Raw 264.7 cells. **e**, **f** Confocal images of MTR transfer from mito-depleted to WT cells (**e**) or WT to mito-depleted cells (**f**) (Raw 264.7 cell, red arrow indicated donor, green arrow indicated recipient).

To provide further evidence regarding the equivalence of dye and HMT, we employed Parkin-induced mitochondria-depleted cells^46^ to assess dye transfer. We overexpressed Parkin protein in Raw 264.7 cell and treated with CCCP for 72 h, then we examined the content of mitochondrial DNA (mtDNA) and the protein levels of mitochondrial located proteins, such as pyruvate dehydrogenase (PDHA), fumarate hydratase (FH), citrate synthase (CS), and translocase of outer mitochondrial membrane 20 (TOM20) in CCCP-treated cells. Both qPCR, WB analyses, and immunofluorescence staining revealed that Parkin-induced mitophagy eliminated the vast majority of mitochondria (Fig. 3a–c and Supplementary Fig. 4A). Furthermore, we employed transmission electron microscopy (TEM) to scrutinize the morphology of mitochondria in these cells. The TEM images confirmed that CCCP-treated cells had undergone a significant loss of mitochondria, with a noticeable absence of healthy mitochondria exhibiting cristae, in sharp contrast to untreated cells that displayed robust and healthy mitochondrial structures (Fig. 3d). We proceeded to employ mito-depleted cells as both MTR-labeled donor cells and CFSE-labeled recipient cells, which were co-cultured with wild-type (WT) cells. Confocal images demonstrated the transfer of MTR signal between mito-depleted cells and WT cells (Fig. 3E, F). Notably, neither the donor nor recipient mito-depleted cells exhibited clear mitochondrial morphology compared to the WT cells.

### Mitochondrial dye can transfer from RBCs to recipient cells

**Fig. 4:**
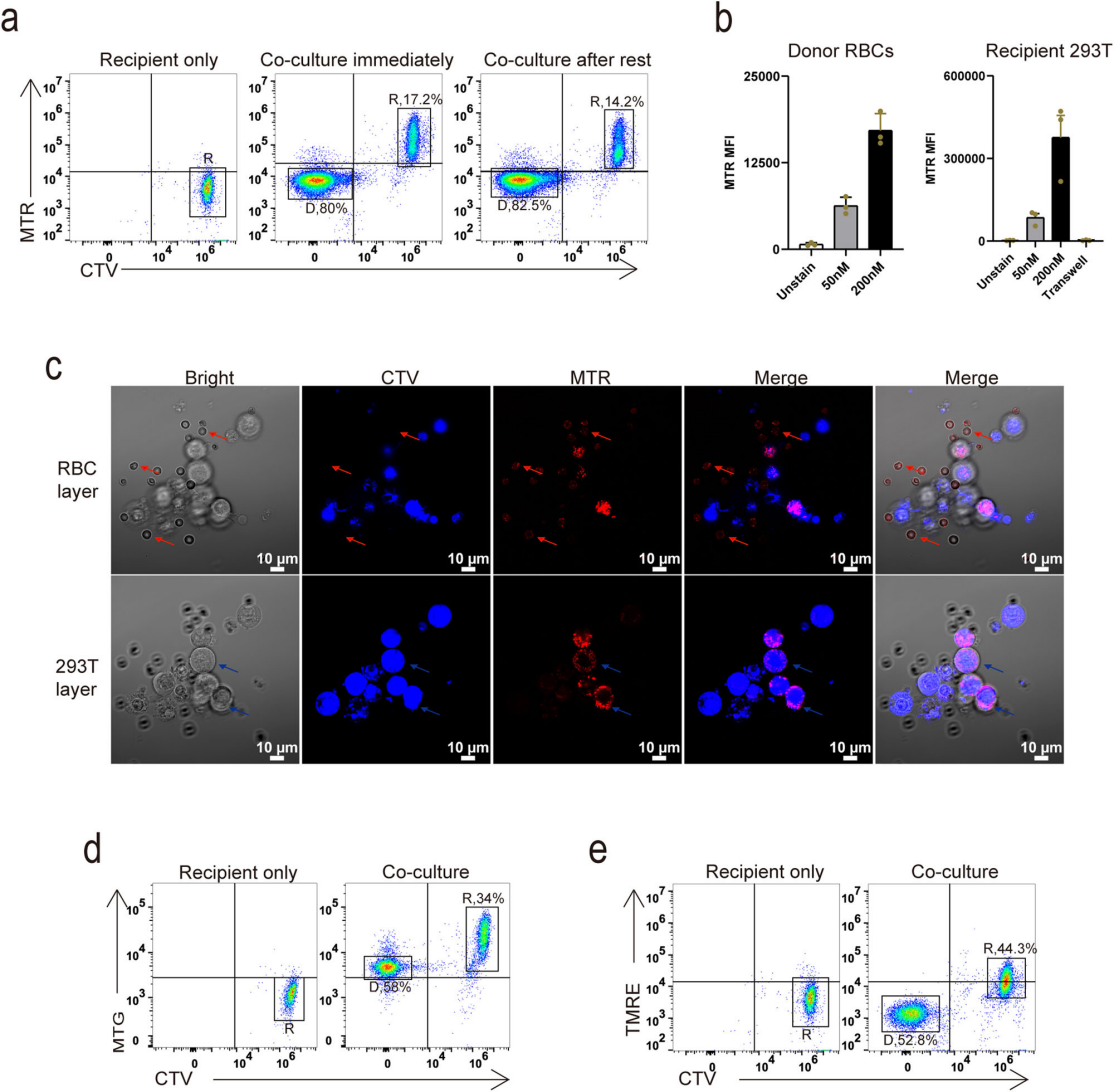
Mitochondria dye can transfer from RBCs to recipient cells. **a** Cytometry analysis of recipient 293 T cells received MTR signal from RBCs. **b** Cytometry analysis of MTR signals of donor RBCs and co-cultured recipient 293 T cells (*n* = 3 biologically independent experiments). **c** Confocal images showed MTR dye transfer from RBCs (red arrow) to recipient 293 T cell (blue arrow). **d**, **e** Cytometry analysis of recipient 293 T cells received MTG signal (**d**) or TMRE signal (**e**) from donor RBC cells. (D, donor; R, recipient).

Considering the potential limitations of Parkin-induced mitophagy, which may not completely eliminate all mitochondria^46^, we sought to verify our hypothesis using cells completely devoid of mitochondria. Remarkably, mature red blood cells (RBCs), which naturally lack mitochondria, proved to be suitable for our investigation. We collected RBCs from mouse peripheral blood and examined the purity of purified RBCs by flow cytometry. Cytometry analysis confirmed that only RBCs, not immune cells, were present in our preparation using the erythrocyte marker TER119 (Supplementary Fig. 3A). We also conducted a western blot analysis by probing for a mitochondrial matrix protein, pyruvate dehydrogenase kinase 1 (PDHK1), to affirm the absence of mitochondrial contamination in the red blood cell (RBC) preparation (Supplementary Figs. 3B, 4B). Subsequently, we labeled RBCs with MTR and co-cultured them with CTV-labeled 293T cells, which demonstrated the transfer of MTR signal from RBCs to recipient 293T cells (Fig. 4A). In some studies, donor cells are labeled with MTR and left in the medium for a few hours before co-cultivation with recipients to mitigate the impact of MTR leakage^18,47^. Similarly, we rested MTR labeled RBCs in medium alone for 4 h and subsequently co-cultured them with recipient 293T cells, which resulted in MTR signal delivery from RBCs to recipient 293T cells (Fig. 4a). The higher the MTR concentration of the donor cells, the stronger the MTR signal received by the recipient cells, and MTR signal transfer depends on cell–cell contact (Fig. 4b). Surprisingly, MTR signal was also detected in RBCs when the MTR concentration increased (Fig. 4b and Supplementary Fig. 3C). Also, when we co-cultured RBCs with CTV labeled recipients 293T cells, the confocal images demonstrated the presence of MTR signal in RBCs, exhibiting fuzzy cell morphology rather than mitochondria, while recipient cells received MTR signal displaying clear mitochondrial morphology (Fig. 4c and Supplementary Fig. 3D). We tested other mitochondria dyes, including Mitotracker Green and TMRE. Mitotracker Green (MTG), another frequently used carbocyanine-based probe, yielded similar results, with MTG also being transfer from RBCs to recipient cells (Fig. 4d and Supplementary Fig. 3E). Interestingly, while TMRE signal was undetectable in RBCs, it was still capable of transfer from RBCs to recipient cells (Fig. 4e and Supplementary Fig. 3F). Collectively, these findings strongly support the notion that mitochondria dye transfer is not equivalent to mitochondria transfer.

## Discussion

Our study aims to shed light on the reliability and specificity of employing mitochondrial dyes, such as Mitotracker, as indicators of HMT. While reports demonstrated HMT with protein and genetic evidence (Supplementary Data 2), our findings underscore the need for caution when interpreting previous literature (Supplementary Data 1). The evidence from proteins, genetic labels, and mitochondrial dyes shows partial alignment, indicating that dyes have some utility but should be used cautiously in studying horizontal mitochondrial transfer. One crucial aspect of our study was the comparison between mitochondrial dye transfer and the actual transfer of mitochondria. Interestingly, previous investigations by Levoux and Lampinen did not directly compare and note the difference between dyes and proteins. However, their results were consistent with ours, despite using different cell types in assessing mitochondrial transfer (Fig. 2)^12,21^. This suggests that the transfer of mitochondria may occur at a much lower frequency than initially presumed based solely on dye-based tracking methods. Consequently, the implications of functional experiments conducted based on mitotracker staining and similar dyes demand scrutiny. Considering the non-specificity of Mitotracker, it is plausible that additional unreported factors exchanged between cells might be responsible for the observed changes in cellular function.

Interestingly, we observed that TMRE failed to produce a fluorescent signal in RBCs (Supplementary Fig. 3F). TMRE, a rhodamine-based probe, is cationic dyes that accumulate in healthy mitochondria-dependent on MMP. When MMP decreased, TMRE escaped into the cytosol, resulting in a very weak fluorescence signal. Consistent with our findings, we observed that TMRE can be transferred from RBCs to recipient cells, but it fails to generate a fluorescent signal within the RBCs themselves (Fig. 4E and Supplementary Fig. 3F). This result showed that mitochondrial dye was indeed present in RBCs in a manner that does not manifest as a visible signal, further corroborating the non-specific nature of mitochondrial dyes .

In conclusion, our study highlights the non-specific nature of mitochondrial dyes and raises important considerations for the interpretation of previous literature about HMT. While mitochondria can indeed transfer between cells, the reliance on mitochondrial dyes such as Mitotracker requires cautious interpretation. The low efficiency of HMT, coupled with the non-specificity of Mitotracker and similar dyes, prompts a reevaluation of previous conclusions. Moving forward, a more comprehensive understanding of the factors contributing to HMT is warranted, encompassing potential non-mitochondrial cargo and mechanisms that underlie genuine HMT.

## Methods and material

### Mice

Wild-type C57BL/6 male mice aged 8–12 weeks were sacrificed to obtain bone marrow for further BMDM differentiation. All mice were housed in specific pathogen-free conditions at Xiamen University Laboratory Animal Center. Institutional Animal Care and Use Committee approved all mouse experiments defined by the Xiamen University Laboratory Animal Center. We have complied with all relevant ethical regulations for animal use.

### Cell culture

All cells were cultured in Dulbecco’s Modified Eagle Medium (DMEM, Sigma) with 10% fetal bovine serum (FBS, Excell) and 100 units/ml of penicillin-streptomycin. Cells were incubated at 37 °C with 5% CO_2_. Mito-depleted cells were generated by adding CCCP (20 μM, MCE) into the culture medium and changing fresh medium containing new CCCP (20 μM) every 12 h until 3 days. For MSC, mouse epididymal adipose tissue were cut into pieces and digested with 0.125% collagenase I for 2 h at 37 °C, then centrifuged at 1500 rpm for 5 min, re-suspend pellet, and culture cells in DMEM. After 3 days culture, remove supernatant and replenish fresh medium containing lentivirus encoding COX8a-GFP into culture dish. Then MSC cells overexpressing COX8a-GFP were digested to co-culture with recipient cells and subsequent Flow cytometry analysis.

In the transwell assay, donor cells were seeded in the upper layer, while recipient cells were seeded in the bottom layer. The transwell has a pore size of 3 μm.

### Plasmid construction and lentivirus production

COX8a-GFP or TOM20-GFP sequence were cloned into lentivirus backbone plasmid (PLV) for further transfection. For COX8a-GFP, the COX8a located sequence were fused with GFP. For TOM20-GFP, PCR amplified whole TOM20 CDS region were fused with GFP. COX8a located sequence and TOM20 PCR primers were shown below:

COX8a located sequence : ATGTCCGTCCTGACGCCGCTGCTGCTGCGGGGCTTGACAGGCTCGGCCCGGCGGCTCCCAGTGCCGCGCGCCAAG;

Primer for TOM20, F : TCTAGAGAATTCGGATCCATGGTGGGCCGGAACAGCGCCATCG; R : CTTTGCAGCTGCCTCCTTTGCAGCTGCCTCTTCCACATCATCTTCAGCCAAGCTCT;

For lentivirus production, a plasmid encoding COX8a-GFP or TOM20-GFP were co-transfected with PMD2G and PSPAX into 293T cells. Harvest supernatant containing lentivirus for further infection.

### Cell staining

Donor cells were stained Mitotracker Red (#M7512, Thermofisher), Mitotracker Green (#M7514, Thermofisher), and TMRE (#601283, Cayman chemical company) with different concentrations at 37 °C for 30 min, recipient cells were staining CTV(5 μM, 20 min) or CFSE(2.5 μM, 5 min) at 37 °C, then both donor cells and recipient cells were washed five times with complete medium. For ruling out dye leakage assay, washed buffer were filtered with a 0.22 filter to remove residual cells for further co-cultivation.

### RBCs isolation

Collecting and isolating C57BL/6 mouse peripheral blood using FICOLL at 1500 rpm, 20 min. The bottom RBCs layer was collected and further purified with biotin-TER119 antibody (#116204, Biolegend). PBMC layer was collected and incubated with ACK for 5 min to remove residual RBCs.

### Fluorescence microscopy

For assays of WT cells co-culture with mito-depleted cells and RBCs co-culture with recipients cells, images were taken by LSM 880 (Zeiss) after cell co-cultivation for 4 h. For video, images were recorded by Cell Discovery 7 (Zeiss) at the beginning of cell co-culture.

### Transmission electron microscopy

WT and mito-depleted Raw 264.7 cells were collected into a 2 ml EP tube, after which cells were washed in PBS and fixed in 2.5% GA at 4 °C overnight. Cells were washed three times with fresh PB solution, then, discarded PB solution and added osmic acid at 4 °C for 2 h, after which cells were washed three times in PB solution within 15 min at room temperature. Samples were dehydrated in a gradient ethanol series (30, 50, 70, 90, 100%), each for 15 min. Samples were deethanolized in a gradient acetone series (25, 50, 75, 100%), each for 15 min. Samples were deacetone in a gradient resin series (25, 50, 75, 100%), each for 2 h. Then, samples were embedded in Epon resin. Embedded samples were polymerized at 70 °C for 24 h. Finally, samples were cut into 60 nm and observed using a Hitachi HT-7800 electron microscope.

### Flow cytometry

Co-cultured cells at different time points were collected and filtered with a 300-um mesh filter. Samples were subjected to flow cytometry using Cytoflex (Beckman). Fluorescence-labeled antibodies used in the current study: Percp conjugated anti-CD45 (#45-0451-82, Invitrogen) and biotin-conjugated anti-TER119 (#116204, Biolegend).

### Western blotting

Cells were lysed in RIPA buffer (50 mM Tris-HCI, 150 mM Nacl, 0.1% SDS, 1.5% NP40, 0.5% deoxycholate, 2 mM Mgcl_2_) containing proteinase inhibitor for 30 min at 4 °C, then boiled in the SDS-PAGE loading buffer (P0015, Beyotime). Equal amounts of protein from each sample were subject to 10% SDS-PAGE and immunoblot analysis. Antibody used in current study includes anti-pyruvate dehydrogenase (PDHA, #3205, Cell signaling technology, 1:1000), anti-fumarate hydratase (FH, #4567, Cell signaling technology, 1:1000), anti-citrate synthase (CS, #14309, Cell signaling technology, 1:1000), anti-pyruvate dehydrogenase kinase 1 (PDHK1, #3820, Cell signaling technology, 1:1000), anti-TOM20 (#11802-1-AP, Proteintech, 1:1000), and anti- Actin (#AC026, Abclonal, 1:1000).

### Quantitative real-time PCR

Mito-depleted cell’s genome were extracted by genome extracted kit (DP304, Tiangen). Real-time PCR were carried out using SYBR master mix (#RM21203, abclonal). Mitochondria genome content was normalized to the nuclear genome content. Specific primers in the current study were shown below :

nuclear genome primer,

B2M-F : ATGGGAAGCCGAACATACTG,

B2M-R : CAGTCTCAGTGGGGGTGAA;

mitochondrial genome primer,

mMitoF : CTAGAAACCCCGAAACCAAA,

mMitoR : CCAGCTATCACCAAGCTCGT.

### Supplementary video. Mitotracker Red transfer from iBMDM donor to recipient cells

The red box indicated MTR transfer from MTR-labeled donor cells (red staining) to contacted CFSE-labeled recipient cells (blue staining); the Blue box indicated recipients cell alone can not obtain MTR from donor cells.

### Statistics and reproducibility

In the present study, all experiments were basically conducted using at least two independent experiments, each containing one replicate. For co-culture experiments, over 200,000 cells were seeded in a 24-well plate, and more than 30,000 cells were recorded by flow cytometry (defined as one replicate). For mito-depleted experiments, CCCP-treated cells in a 6 cm dish were collected and analyzed by qPCR, WB, and TEM (defined as one replicate). Graphs were made, and statistical analyses were performed using Prism software. Data were shown as means ± SD (error bars). Two-tailed unpaired *t*-tests were performed.

### Reporting summary

Further information on research design is available in the Nature Portfolio Reporting Summary linked to this article.

### Supplementary information

Supplementary information

Description of Supplementary Materials

Supplementary Data 1

Supplementary Data 2

Supplementary Data 3

Supplementary Video

Reporting Summary

## Data Availability

The sequence of a primer used in plasmids construction and qPCR were listed in Methods. All of the uncropped images in western blotting were shown in Supplementary Fig. 4. The source data of flow cytometry, transmission electron microscopy, and graphs were shown in supplementary Data 3.
