# Supplementary material for: Exploring the limitations of mitochondrial dye as a genuine horizontal mitochondrial transfer surrogate
Source: Commun Biol. 2024 Mar 7;7:281. doi: 10.1038/s42003-024-05964-6 (PMC10917768; doi:10.1038/s42003-024-05964-6)
Supplement: Supplementary file 1 — Supplementary information [file 42003_2024_5964_MOESM1_ESM.pdf]

Supplementary Information for

Exploring the Limitations of Mitochondrial Dye as a Genuine Horizontal Mitochondrial Transfer Surrogate

Chuanfang Chen*, Haige Li, Jia Zhang, Shih-Chin Cheng*

*Corresponding authors

E-mail: Shih-Chin Cheng : jamescheng@xmu.edu.cn;

Chuanfang Chen : 21620200156475@stu.xmu.edu.cn

This PDF file includes:

Supplementary Figure 1-4

Other supplementary materials for this manuscript include the following:

File name: Supplementary Data 1

Description: Overview of mitochondria dye staining involved in HMT in this study.

File name: Supplementary Data 2

Description: Overview of protein or genetic evidence involved in HMT in this study.

File name: Supplementary Data 3

Description: Source data presented in this study.

File name: Supplementary Video

Description: Video shows MR transfer.

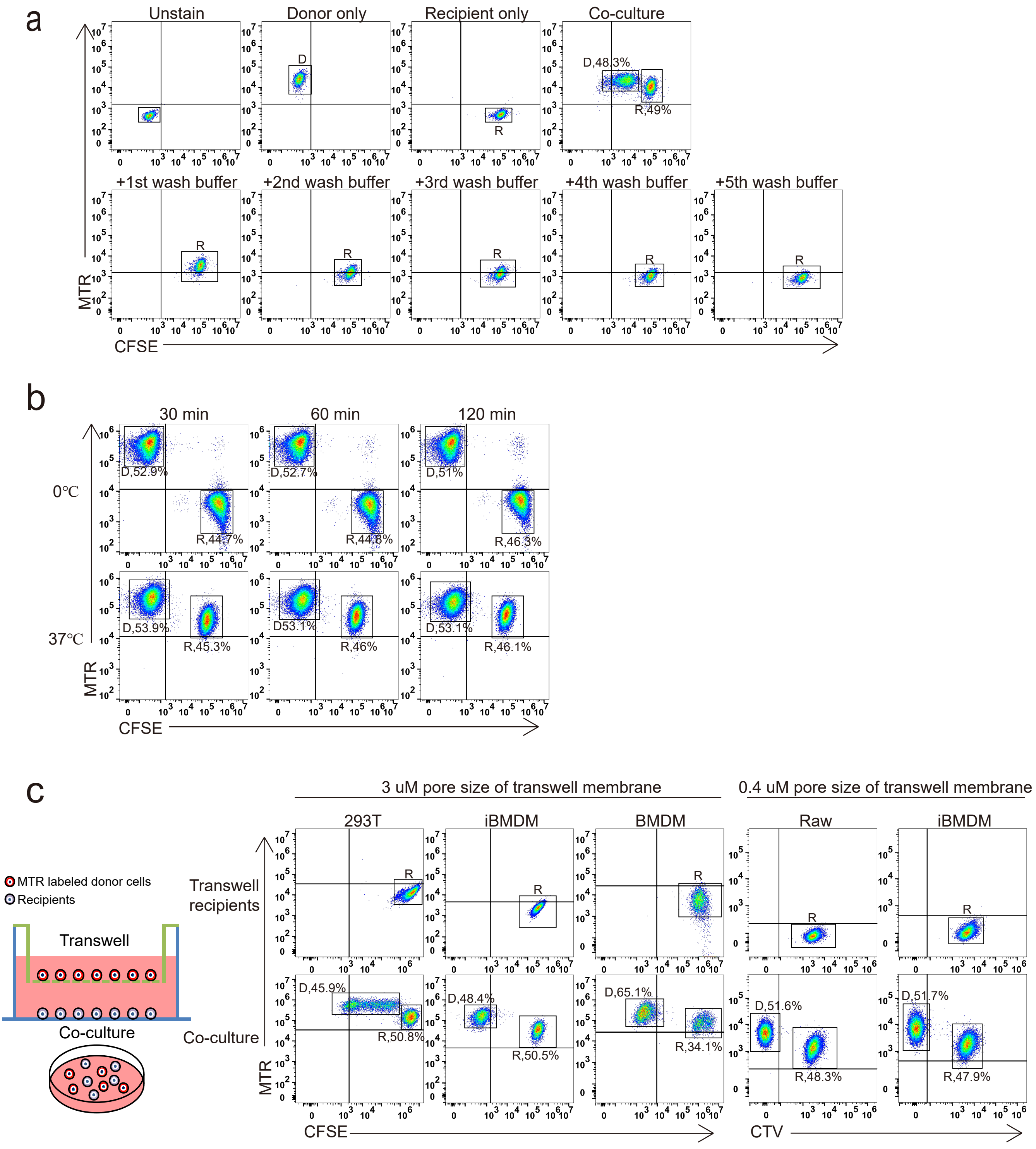

Supplementary Figure 1. Mitochondrial dye can transfer between cells. (**a** Cytometry analysis of recipient iBMDM cells received MTR signal when co-cultured with washed buffer. (**b**) Cytometry analysis of recipient iBMDM cells received MTR signal on different temperature at different times. (**c**) Cytometry analysis of recipient iBMDM cells received MTR signal at co-culture or transwell condition. (D, donor; R, recipient)

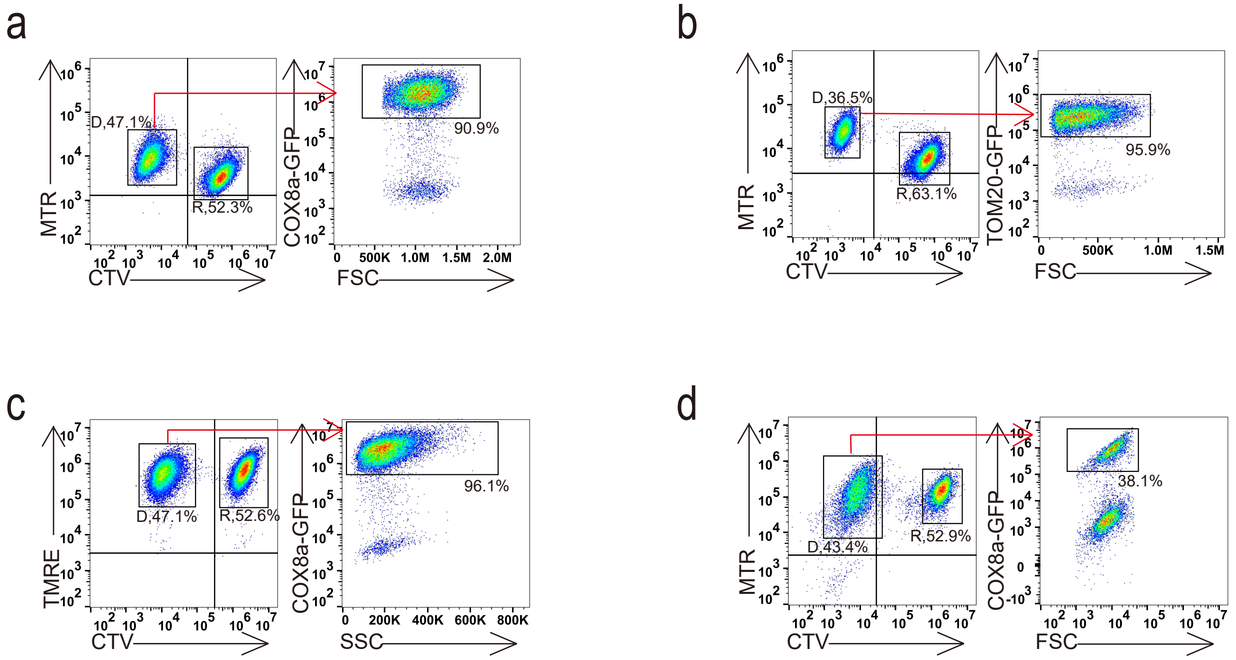

Supplementary Figure 2. The proportion of mitochondria target GFP expression in donor cells. Fig S2a-S2d referred to **Fig 2a-2d** donor cells respectively.

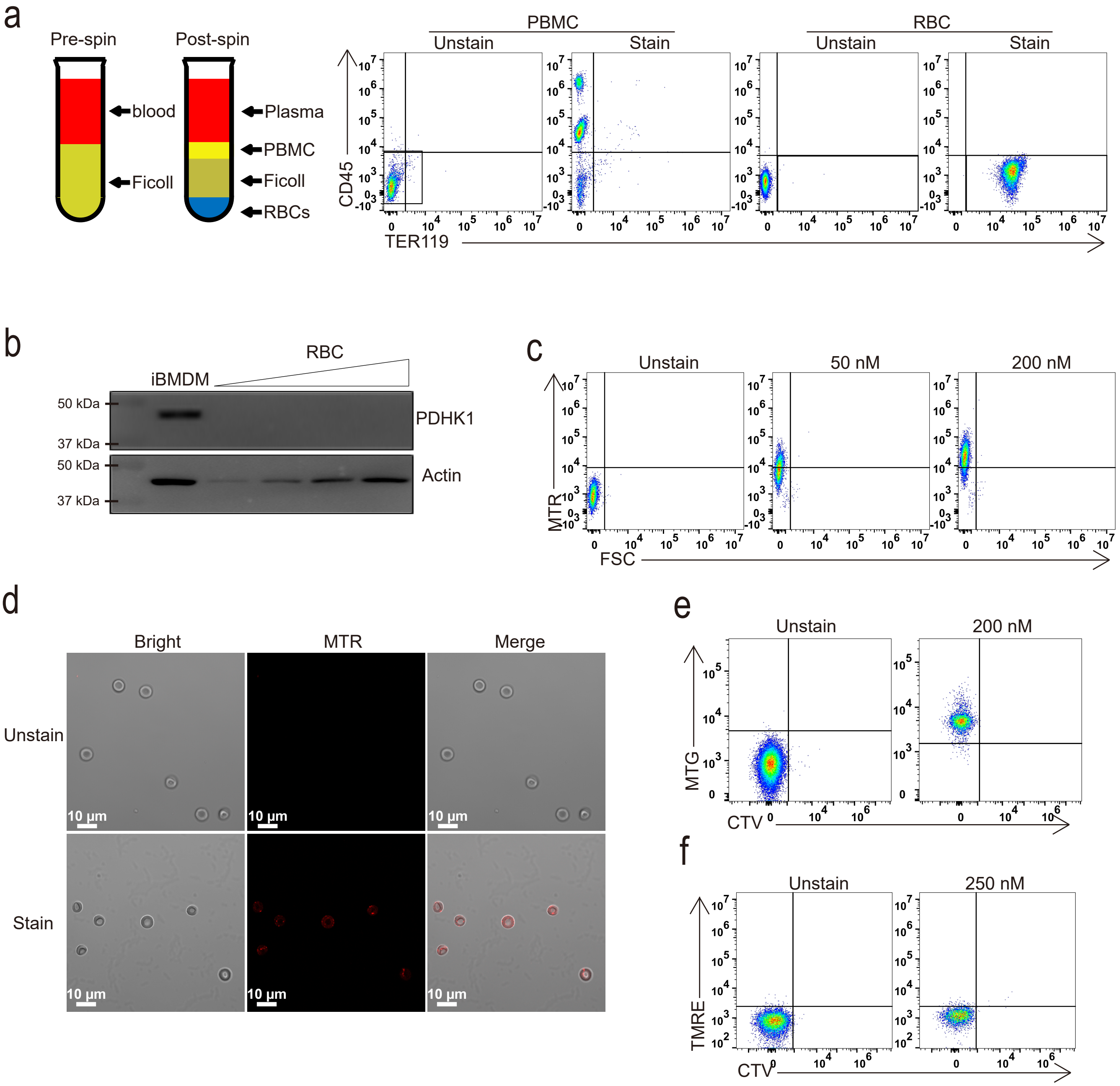

Supplementary Figure 3. Mitochondrial dye transfer was not equal to mitochondria transfer. (**a**) Purity of RBCs collected from Ficoll isolation. (**b**) Western blot analysis of PDHK1 in iBMDM cell (as control) and RBCs. Blots were tested from separated gels loading the same volume of sample. (**c**)Cytometry analysis of RBCs MTR signal. (**d**) Confocal images showed MTR can stain RBCs exhibiting red fluorescence. (**e** and **f**) Cytometry analysis of donor RBCs labeled with Mitotracker Green (**e**) or TMRE (**f**).

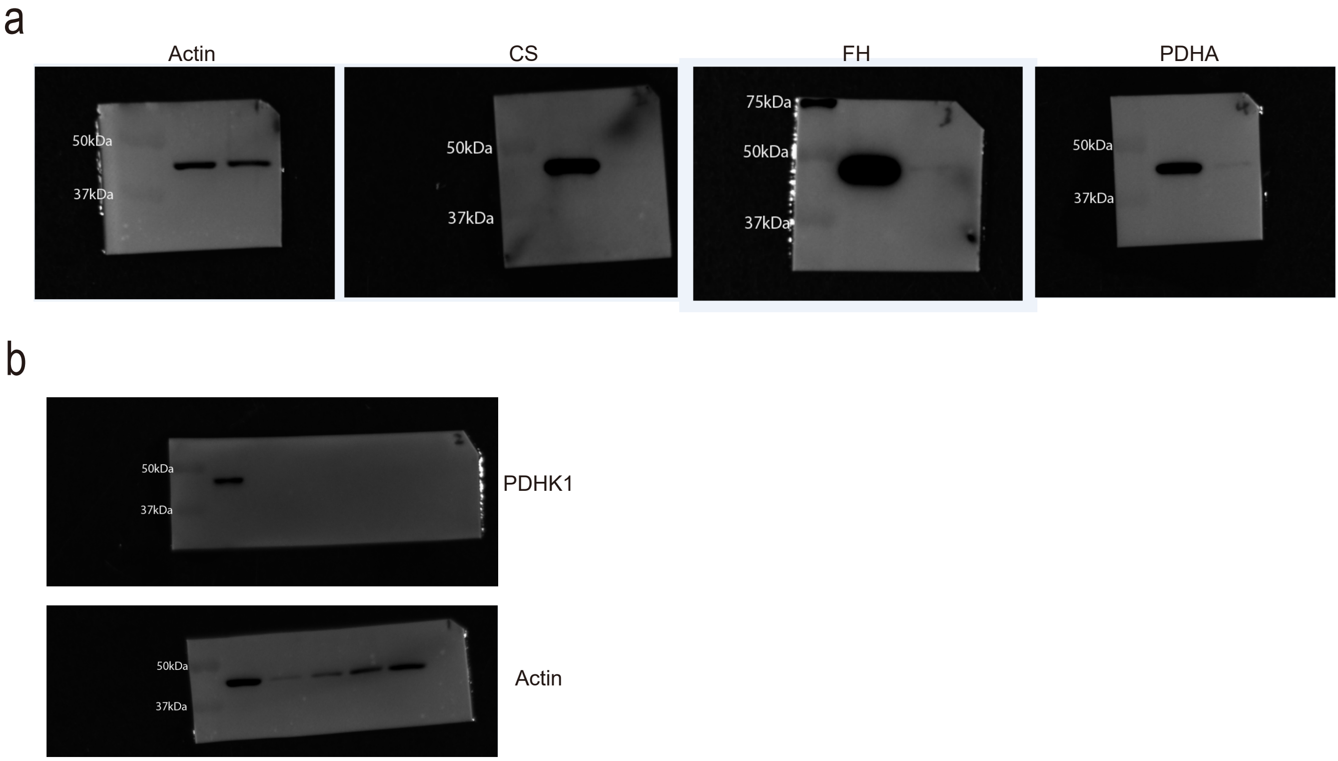

Supplementary Figure 4. Unedited blots for Fig 3b and supplementary Figure 3b. Fig S4a-S4b referred to **Fig 3b** and **Fig S3b** respectively. Blots were tested from separated gels loading the same volume of sample.
