# Supplementary material for: Exploring the limitations of mitochondrial dye as a genuine horizontal mitochondrial transfer surrogate
Source: Commun Biol. 2024 Mar 7;7:281. doi: 10.1038/s42003-024-05964-6 (PMC10917768; doi:10.1038/s42003-024-05964-6)
Supplement: Supplementary file 2 — Description of Supplementary Materials [file 42003_2024_5964_MOESM2_ESM.docx]

**Description of Additional Supplementary Files**

**File name:** Supplementary Data 1

**Description:** Overview of mitochondria dye staining involved in HMT

**File name:** Supplementary Data 2

**Description:** Overview of protein or genetic evidence involved in HMT

**File name:** Supplementary Data 3

**Description:** source data presented in this study.

**File name:** Supplementary Video

**Description:** Video showing MR transfer
