# Supplementary material for: Exploring the limitations of mitochondrial dye as a genuine horizontal mitochondrial transfer surrogate
Source: Commun Biol. 2024 Mar 7;7:281. doi: 10.1038/s42003-024-05964-6 (PMC10917768; doi:10.1038/s42003-024-05964-6)
Supplement: Supplementary file 3 — Supplementary Data 1 [file 42003_2024_5964_MOESM3_ESM.pdf]

Supplementary Data 1. Overview of mitochondria dye staining involved in HMT

Donor cell	Acceptor cell	Reference	PMID	Experimental model	Methods to indicate HMT
Rat neonatal cardiomyocytes	Human endothelial progenitor cells	Koyanagi et al., 2005	15879310	in vitro	MitoTracker
Macrophage	Macrophage	Onfelt et al., 2006	17142745	in vitro	MitoTracker
Mesenchymal stem cells	Rat cardiac myocytes	Plotnikov et al., 2008	18088382	in vitro	TMRE/MitoTracker
Rat mesenchymal stem cells	Rat lung endothelial cells	Otsu et al., 2009	19036701	in vitro	MitoTracker
Mouse endothelial progenitor cells	Human umbilical vein endothelial cells	Yasuda et al., 2010	20167859	in vitro	MitoTracker
Mesenchymal stem cells	Cardiomyoblasts	Cselenyak et al., 2010	20406471	in vitro	MitoTracker
Rat renal tubular cells	Mesenchymal stem cells	Plotnikov et al., 2010	20599955	in vitro	MitoTracker/JC-1
Mesenchymal stem cells	Rat renal tubular cells				
Human adipose-derived stem cells	Mouse cardiomyocytes	Acquistapace et al., 2011	21433223	in vitro	MitoTracker
Rat ventricular cardiomyocytes	Rat fibroblasts	He et al., 2011	21719573	in vitro	MitoTracker
Rat fibroblasts	Rat fibroblasts				
Human proximal tubular epithelial cells	Human proximal tubular epithelial cells	Domhan et al., 2011	21738629	in vitro	MitoTracker
Human pleural mesothelioma cells	Human pleural mesothelioma cells	Lou et al., 2012	22427958	in vitro	MitoTracker
Human retinal pigment epithelial cells	Human retinal pigment epithelial cells	Witting et al., 2012	22457742	in vitro	JC-1
Mesenchymal stem cells	Human vascular smooth muscle cells	Vallabhaneni et al., 2012	22676452	in vitro	MitoTracker
Human vascular smooth muscle cells	Mesenchymal stem cells				
Mesenchymal stem cells	Human ovarian cancer cells	Pasquier et al., 2013	23574623	in vitro	MitoTracker
Mesenchymal stem cells	Human breast cancer cells				
Endothelial cells	Human ovarian cancer cells				
Endothelial cells	Human breast cancer cells				
Mesenchymal stem cells	Lung epithelial cells	Li et al., 2014	24738760	in vitro	MitoTracker
Platelets	Neutrophils	Boudreau et al., 2014	25082876	in vitro	MitoTracker
Rat pheochromocytoma cells	Rat pheochromocytoma cells	Wang et al., 2015	25571977	in vitro	TMRM/MitoTracker/mito-DsRed
Mesenchymal stem cells	Human 143B osteosarcoma ρ 0 cell	Lin et al., 2015	25746175	in vitro	MitoTracker
Mesenchymal stem cells	Rat cardiomyoblasts	Han et al., 2016	26718099	in vitro	MitoTracker
Mesenchymal stem cells	Lung alveolar macrophages	Jackson et al., 2016	27059413	in vitro/in vivo	MitoTracker
Bone marrow stromal cells	Acute myeloid leukemic cells	Moschoi et al., 2016	27257182	in vitro/in vivo	MitoTracker/OMI-HTRA2/PCR
Astrocytes	Neuronal cells	Hayakawa et al., 2016	27466127	in vitro & in vivo	MitoTracker
Induced pluripotent stem cell derived mesenchymal stem cells	Cardiomyocyte	Zhang et al., 2016	27641650	in vitro & in vivo	MitoTracker/mito-GFP/PCR
Malignant urothelial carcinoma cells	Non-malignant urinary papillary urothelial tumor cells	Lu et al., 2017	28107184	in vitro	MitoTracker
Mesenchymal stem cells	Human cardiomyocytes	Mahrouf-Yorgov et al., 2017	28524859	in vitro	MitoTracker
Mesenchymal stem cells	Human endothelial cell				
Human cardiomyocytes	Mesenchymal stem cells				
Human endothelial cell	Mesenchymal stem cells				
Mesenchymal stem cells	Mesenchymal stem cells	Li et al., 2017	28596814	in vitro	MitoTracker

Mesenchymal stromal cells	Macrophages	Morrison et al., 2017	28598224	in vitro	MitoTracker
Mesenchymal stem cells	Myoclonus epilepsy with ragged-red fiber cybrid cells	Chuang et al., 2017	28607632	in vitro	MitoTracker/mito-GFP/mito-RFP
Bone marrow stromal cells	Acute myeloid leukemia cells	Marlein et al., 2017	28733324	in vitro/in vivo	MitoTracker/mito-mcherry/PCR
Human stressed astrocytes	Human healthy astrocytes	Rostami et al., 2017	29089438	in vitro	MitoTracker
Human healthy astrocytes	Human stressed astrocytes	Rostami et al., 2017	29089438	in vitro	MitoTracker
Monkey kidney cells	Monkey kidney cells	Guo et al., 2018	29307597	in vitro	TMRM
Porcine alveolar macrophages	Porcine alveolar macrophages	Guo et al., 2018	29307597	in vitro	TMRM
Porcine umbilical cord mesenchymal stem cells	Porcine alveolar macrophages	Guo et al., 2018	29307597	in vitro	TMRM
Renal scattered tubular-like cells	Tubular epithelial cells	Zou et al., 2018	29352176	in vitro	MitoTracker
T cell acute lymphoblastic leukemia cells	Mesenchymal stem cells	Wang e al., 2018	29357914	in vitro	MitoTracker
Mesenchymal stem cells	T Cell acute lymphoblastic leukemia cells	Wang e al., 2018	29357914	in vitro	MitoTracker
Cardiac myofibroblasts	Cardiomyocytes	Shen et al., 2018	29362447	in vitro	MitoTracker/mito-GFP
Myeloid-derived regulatory cells	T Cells	Hough et al., 2018	29986209	in vitro	MitoTracker/mito-GFP
Human induced pluripotent stem cell–derived MSCs	Human bronchial epithelium cells	Yao et al., 2018	30344008	in vitro/in vivo	MitoTracker/mito-GFP
Human induced pluripotent stem cell–derived MSCs	Murine epithelial cells	Yao et al., 2018	30344008	in vitro/in vivo	MitoTracker/mito-GFP
Endothelial progenitor cells	Neurons	Borlongan et al., 2019	30375940	in vivo	MitoTracker
Mesenchymal stem cells	Rat cardiomyocytes	Palowal et al., 2018	30409230	in vitro	MitoTracker
Mesenchymal stem cells	Human astroblastoma cells	Palowal et al., 2018	30409230	in vitro	MitoTracker
Bone marrow stromal cells	Multiple myeloma cells	Marlein et al., 2019	30622116	in vitro & in vivo	MitoTracker/PCR/mito-mcherry
Cancer-associated fibroblasts	Prostate cancer PC3 cells	Ippolito et al., 2019	30936458	in vitro/in vivo	MitoTracker/mito-DsRed/GFP
Cancer-associated fibroblasts	Prostate cancer DU145 cells	Ippolito et al., 2019	30936458	in vitro/in vivo	MitoTracker/mito-DsRed/GFP
Astrocytes	Primary rat neuronal cells	Lippert and Borlongan, 2019	30972972	in vitro	MitoTracker
Mesenchymal stem cells	Neurons	Li et al., 2019	31037154	in vitro	MitoTracker
Mesenchymal stem cells	Peripheral blood mononuclear cells	Luz-Crawford et al., 2019	31370879	in vitro	MitoTracker
Epithelial cells	Acute lymphoblastic leukemia cells	Burt et al., 2019	31501154	in vitro	MitoTracker/PCR
Mesenchymal stem cells	Acute lymphoblastic leukemia cells	Burt et al., 2019	31501154	in vitro	MitoTracker/PCR
Fibroblasts	Fibroblasts	Jin et al., 2019	31703695	in vitro	MitoTracker
CD133+ Scattered tubular cells	CD133- Tubular cells	Zou et al., 2020	31759629	in vitro	MitoTracker
Mesenchymal stem cells	Peripheral blood mononuclear cell	C-Court et al., 2020	31984629	in vitro & in vivo	MitoTracker/PCR
Bone marrow derived mesenchymal stem cells	Macrophage RAW264.7 cell	Zhou et al., 2020	32247615	in vitro	MitoTracker
Astrocyte	Neuroblastoma cell	Wang et al., 2020	32460411	in vitro	MitoTracker
Mesenchymal stem cells	Neurons	Tseng et al., 2021	32501156	in vitro	MitoTracker
Bone marrow mesenchymal stromal cells	Hematopoietic cells	Golan et al., 2020	32929449	in vitro & in vivo	MitoTracker/mito-Dendra2
Hematopoietic cells	Bone marrow mesenchymal stromal cells	Golan et al., 2020	32929449	in vitro & in vivo	MitoTracker/mito-Dendra2
Tumor-activated stromal cell	Glioblastoma	Salaud et al., 2020	32943183	in vitro	MitoTracker
Platelets	Mesenchymal stem cells	Levoux et al., 2021	33400911	in vitro	MitoTracker/DsRed
Human glioblastoma cells	Human primary astrocytes	Valdebenito et al., 2021	34267246	in vitro	MitoTracker

Bone marrow stromal cells	Myeloma cells	Matula et al., 2021	34298674	in vitro	MitoTracker
Myeloma cells	Bone marrow stromal cells				
Human mesenchymal stem cells	Injured alveolar epithelial cells	Huang et al., 2021	34586849	in vitro & in vivo	MitoTracker/PCR
Murine high-metastatic lung carcinoma cells	Murine low-metastatic lung carcinoma cells	Takenaga et al., 2021	34615464	in vitro & in vivo	MitoTracker/LT-Red
Murine low-metastatic lung carcinoma cells	Murine high-metastatic lung carcinoma cells				
Murine high-metastatic lung carcinoma cells	Murine low-metastatic lung carcinoma cells and cancer-associated fibroblasts				
Effector immune cells	Breast cancer cells	Saha et al., 2022	34795441	in vitro	MitoTracker
T cells	Lung carcinoma cells				
T cells	Melanoma cells				
Macrophage	Neuronal cell	Vlist et al., 2022	34921782	in vitro & in vivo	MitoTracker/mito-Dendra2
Adipocytes	Macrophage	Rosina et al., 2022	35305295	in vitro	MitoTracker
Human neurons	Human astrocytes	Lampinen et al., 2022	35569719	in vitro	MitoTracker/mito-GFP/mcherry
Murine neurons	Murine astrocytes				
Macrophages	Mesenchymal stem cells	Cai et al., 2023	36507570	in vitro/in vivo	MitoTracker/mito-GFP
Mesenchymal stem cells	T cell	Akhter et al., 2023	36694226	in vitro	MitoTracker
Mesenchymal stem cells	Tenocytes	Wei et al., 2023	37101277	in vitro/in vivo	MitoTracker
Microglia	Neuronal cell	Chakraborty et al., 2023	37202391	in vitro	MitoTracker
Lymphocytes	Mouse lung cancer cell	Wang et al., 2023	37236579	in vitro	MitoTracker
Mesenchymal stem cells	Glioblastoma stem cells	Nakhle et al., 2023	37377608	in vitro	MitoTracker
Human umbilical MSCs	Mouse neuronal cell	Yao et al., 2023	37699320	in vitro & in vivo	MitoTracker/mito-GFP
Human placental-derived MSCs	Mouse lung epithelial cells	Huang et al., 2023	37723135	in vitro	MitoTracker
Human placental-derived MSCs	Human umbilical vein endothelial cells				
Human placental-derived MSCs	Human normal lung fibroblasts				

This table lists the reported horizontal mitochondrial transfer studies, including donor/recipient cells, experimental model and dye staining method.
