# Supplementary material for: Exploring the limitations of mitochondrial dye as a genuine horizontal mitochondrial transfer surrogate
Source: Commun Biol. 2024 Mar 7;7:281. doi: 10.1038/s42003-024-05964-6 (PMC10917768; doi:10.1038/s42003-024-05964-6)
Supplement: Supplementary file 4 — Supplementary Data 2 [file 42003_2024_5964_MOESM4_ESM.pdf]

# Supplementary Data 2. Overview of protein or genetic evidence involved in HMT

| Donor cell | Acceptor cell | Reference | PMID | Experimental model | Methods to indicate HMT |
|---|---|---|---|---|---|
| Human mesenchymal Stem Cells | Human osteosarcoma 143B ρ0 cell | Cho et al., 2012 | 22412925 | in vitro | PCR |
| Bone marrow–derived stromal cells | Mouse alveolar type II cells | Islam et al., 2012 | 22504485 | in vivo | mito-RFP |
| Mesenchymal Stem Cells | Epithelial cells | Ahmad et al., 2014 | 24431222 | in vitro | mito-GFP |
| Ad293 | Ad293 | Lin et al.,2016 | 27165101 | in vitro | mito-GFP |
| Mesenchymal Stem Cells | Corneal epithelial cells | Jiang et al., 2016 | 27831562 | in vitro | mito-GFP |
| Multipotent mesenchymal Stem Cells | Astrocytes | Babenko et al., 2018 | 29562677 | in vitro | mito-GFP |
| Mesenchymal Stem Cells | Neural stem cells | Boukelmoune, | 30541620 | in vitro | mito-GFP/mito-mcherry |
| Human astrocytes | Human astrocytes | Gao et al., 2019 | 31327963 | in vitro | mito-GFP |
| Human neuronal cells | Human astrocytes | | | | |
| Primary calvarial osteocytes | Primary calvarial osteocytes | Gao et al., 2019 | 31799389 | in vitro & in vivo | mito-Dendra2 |
| Mesenchymal stromal cells | Islet β cells | Rackham et al., 2020 | 31912945 | in vivo | mito-GFP |
| Astrocytes | Neurons | English et al., 2020 | 32197663 | in vitro | mito-mcherry |
| Mesenchymal stem cells | Retinal pigment epithelium cells | Jiang et al., 2020 | 32641991 | in vitro | mito-GFP |
| Mesenchymal stem cells | Corneal endothelial cells | | | | |
| Adipocytes | Macrophages | Brestoff et al., 2021 | 33278339 | in vivo | mito-Dendra2 |
| Adipocytes | Macrophage | Borcherding et al., 2022 | 36070756 | in vivo | mito-Dendra2 |
| Immortalized human astrocytes | Human-derived glioblastoma stem-like cells | Watson et al., 2023 | 37169842 | in vitro & in vivo | mito-RFP |

**This table lists the reported horizontal mitochondrial transfer studies, including donor/recipient cells, experimental model and genetic or protein labelled method.**
