# Supplementary material for: Exploring the limitations of mitochondrial dye as a genuine horizontal mitochondrial transfer surrogate
Source: Commun Biol. 2024 Mar 7;7:281. doi: 10.1038/s42003-024-05964-6 (PMC10917768; doi:10.1038/s42003-024-05964-6)
Supplement: Supplementary file 7 — Reporting Summary [file 42003_2024_5964_MOESM7_ESM.pdf]

nature portfolio

Corresponding author(s): Shih-Chin Cheng COMMSBIO-23-3876

Last updated by author(s): Feb 11, 2024

Reporting Summary

Nature Portfolio wishes to improve the reproducibility of the work that we publish. This form provides structure for consistency and transparency in reporting. For further information on Nature Portfolio policies, see our Editorial Policies and the Editorial Policy Checklist.

Statistics

For all statistical analyses, confirm that the following items are present in the figure legend, table legend, main text, or Methods section.

n/a	Confirmed	
☐	☒	The exact sample size (*n*) for each experimental group/condition, given as a discrete number and unit of measurement
☐	☒	A statement on whether measurements were taken from distinct samples or whether the same sample was measured repeatedly
☐	☒	The statistical test(s) used AND whether they are one- or two-sided<br>*Only common tests should be described solely by name; describe more complex techniques in the Methods section.*
☒	☐	A description of all covariates tested
☒	☐	A description of any assumptions or corrections, such as tests of normality and adjustment for multiple comparisons
☐	☒	A full description of the statistical parameters including central tendency (e.g. means) or other basic estimates (e.g. regression coefficient) AND variation (e.g. standard deviation) or associated estimates of uncertainty (e.g. confidence intervals)
☐	☒	For null hypothesis testing, the test statistic (e.g. *F*, *t*, *r*) with confidence intervals, effect sizes, degrees of freedom and *P* value noted<br>*Give P values as exact values whenever suitable.*
☒	☐	For Bayesian analysis, information on the choice of priors and Markov chain Monte Carlo settings
☒	☐	For hierarchical and complex designs, identification of the appropriate level for tests and full reporting of outcomes
☒	☐	Estimates of effect sizes (e.g. Cohen's *d*, Pearson's *r*), indicating how they were calculated

Our web collection on statistics for biologists contains articles on many of the points above.

Software and code

Policy information about availability of computer code

Data collection	Zeiss Zen (Black Edition) v.3.1<br>CFX96 Touch Real-Time PCR detection system (Bio-rad)<br>CytoExpert<br>BD FACSDiva software
Data analysis	FlowJo V.10<br>GraphPad Prism 8.0.2<br>Microsoft Excel 2010<br>Zeiss Zen (Blue Edition v.3.1)

For manuscripts utilizing custom algorithms or software that are central to the research but not yet described in published literature, software must be made available to editors and reviewers. We strongly encourage code deposition in a community repository (e.g. GitHub). See the Nature Portfolio guidelines for submitting code & software for further information.

Data

Policy information about availability of data

All manuscripts must include a data availability statement. This statement should provide the following information, where applicable:

- Accession codes, unique identifiers, or web links for publicly available datasets
- A description of any restrictions on data availability
- For clinical datasets or third party data, please ensure that the statement adheres to our policy

All data that support the findings are available within the manuscript and the Supplementary Information.

Research involving human participants, their data, or biological material

Policy information about studies with human participants or human data. See also policy information about sex, gender (identity/presentation), and sexual orientation and race, ethnicity and racism.

Reporting on sex and gender	The study did not involve human data.
Reporting on race, ethnicity, or other socially relevant groupings	The study did not involve human data.
Population characteristics	The study did not involve human data.
Recruitment	The study did not involve human data.
Ethics oversight	The study did not involve human data.

Note that full information on the approval of the study protocol must also be provided in the manuscript.

Field-specific reporting

Please select the one below that is the best fit for your research. If you are not sure, read the appropriate sections before making your selection.

☒ Life sciences ☐ Behavioural & social sciences ☐ Ecological, evolutionary & environmental sciences

For a reference copy of the document with all sections, see nature.com/documents/nr-reporting-summary-flat.pdf

Life sciences study design

All studies must disclose on these points even when the disclosure is negative.

Sample size	No statistical methods were used to predetermined sample size. Sample size were chosen based on previous studies which had shown robust statistical power.
Data exclusions	No data were excluded.
Replication	For all staining procedures, a sufficient number of cells was analyzed to control for biological variability between cases. Image analysis was performed at the same time for each experiment.
Randomization	All cells were selected randomly based on dye signal.
Blinding	Blinding was not done for staining experiments analysis as the cells were randomly selected based on dye signal and protein expression was analyzed following image capture which was consistent for all images.

Reporting for specific materials, systems and methods

We require information from authors about some types of materials, experimental systems and methods used in many studies. Here, indicate whether each material, system or method listed is relevant to your study. If you are not sure if a list item applies to your research, read the appropriate section before selecting a response.

Materials & experimental systems

n/a	Involved in the study
☐	☒ Antibodies
☐	☒ Eukaryotic cell lines
☒	☐ Palaeontology and archaeology
☒	☐ Animals and other organisms
☒	☐ Clinical data
☒	☐ Dual use research of concern
☒	☐ Plants

Methods

n/a	Involved in the study
☒	☐ ChIP-seq
☐	☒ Flow cytometry
☒	☐ MRI-based neuroimaging

Antibodies

Antibodies used

anti-Pyruvate Dehydrogenase (PDHA, #3205, Cell signaling technology)
anti-Fumarate Hydratase (FH, #4567, Cell signaling technology)
anti-Citrate Synthase (CS, #14309, Cell signaling technology)
anti-Pyruvate dehydrogenase kinase 1 (PDHK1, #3820, Cell signaling technology)
anti- Actin (#AC026, Abclonal)
Percp conjugated anti-CD45 (#45-0451-82, Invitrogen)
biotin conjugated anti-TER119 (#116204, Biolegend)
anti-Tom20 (11802-1-AP, Proteintech)
Alexa Fluor 488 conjugated secondary antibody (A-21206, Invitrogen)

Validation

All antibodies used were commercially available and validated by the manufacturers and have been extensively used in previous publications . Links to manufacturers' websites that contain relevant references are below.
anti-Pyruvate Dehydrogenase (PDHA, #3205, Cell signaling technology) : https://www.cellsignal.com/products/primary-antibodies/pyruvate-dehydrogenase-c54g1-rabbit-mab/3205
anti-Fumarate Hydratase (FH, #4567, Cell signaling technology) : https://www.cellsignal.cn/products/primary-antibodies/fumarase-d9c5-rabbit-mab/4567
anti-Citrate Synthase (CS, #14309, Cell signaling technology) : https://www.cellsignal.com/products/primary-antibodies/citrate-synthase-d7v8b-rabbit-mab/14309
anti-Pyruvate dehydrogenase kinase 1 (PDHK1, #3820, Cell signaling technology) : https://www.cellsignal.cn/products/primary-antibodies/pdhk1-c47h1-rabbit-mab/3820?site-search-type=Products&N=4294956287&Ntt=3820t&fromPage=plp&_requestid=2477004
anti- Actin (#AC026, Abclonal) : https://abclonal.com.cn/catalog/AC026
Percp conjugated anti-CD45 (#45-0451-82, Invitrogen) : https://www.thermofisher.cn/cn/zh/antibody/product/CD45-Antibody-clone-30-F11-Monoclonal/45-0451-82
biotin conjugated anti-TER119 (#116204, Biolegend) : https://www.biolegend.com/en-us/products/biotin-anti-mouse-ter-119-erythroid-cells-antibody-1864?GroupID=ImportedGROUP1
anti-Tom20 (11802-1-AP, Proteintech) : https://www.ptgcn.com/products/TOM20-Antibody-11802-1-AP.htm
Alexa Fluor 488 conjugated secondary antibody (A-21206, Invitrogen) : https://www.thermofisher.cn/cn/zh/antibody/product/Donkey-anti-Rabbit-IgG-H-L-Highly-Cross-Adsorbed-Secondary-Antibody-Polyclonal/A-21206

Eukaryotic cell lines

Policy information about cell lines and Sex and Gender in Research

Cell line source(s)

B16, Raw 264.7, 293T, MC38 and B16 cell line were gift from professor Han Jiahuai (Colleage of life science, Xiamen university, CHINA). BMDM and mesenchymal stem cells were obtained from male mice bone marrow and epididymis adipose tissue.

Authentication

All cell lines have been extensively used in previous publications.

Mycoplasma contamination

All cell lines were test negative for mycoplasma contamination.

Commonly misidentified lines
(See ICLAC register)

There was no commonly misidentified lines used in the study.

Plants

Seed stocks	The study did not involve plants.
Novel plant genotypes	The study did not involve plants.
Authentication	The study did not involve plants.

Flow Cytometry

Plots

Confirm that:

☒ The axis labels state the marker and fluorochrome used (e.g. CD4-FITC).

☒ The axis scales are clearly visible. Include numbers along axes only for bottom left plot of group (a 'group' is an analysis of identical markers).

☒ All plots are contour plots with outliers or pseudocolor plots.

☒ A numerical value for number of cells or percentage (with statistics) is provided.

Methodology

Sample preparation	Co-cultured cells were digested, then filtered with 48 um mesh to loading tube.
Instrument	BD LSRFortessa and Beckman Cytoflex LX.
Software	BD FACSDiva software were applied in BD LSRFortessa to collect sample data. CytoExpert software were applied in Beckman Cytoflex LX to collect sample data. All flow cytometry data were analyzed with FlowJo v.10.
Cell population abundance	All prepared cell sample contained more than 200000 cells. Abundance of cell population was counted by instrument.
Gating strategy	For all co-cultured assay, single cells were first gated with FSC/SSC. For red blood cells(RBCs) assay, RBCs were defined with negative signal of CD45 and positive signal of TER119. For other assay, recipient and donor cells were separated with different staining signal. CFSE or CTV positive cell were defined as recipient while TMRE or Mitotracker RED positive cell were defined as donor cells.

☒ Tick this box to confirm that a figure exemplifying the gating strategy is provided in the Supplementary Information.
